# Protein *Trans*-Splicing of an Atypical Split Intein Showing Structural Flexibility and Cross-Reactivity

**DOI:** 10.1371/journal.pone.0045355

**Published:** 2012-09-14

**Authors:** Huiling Song, Qing Meng, Xiang-Qin Liu

**Affiliations:** 1 Institute of Biological Sciences and Biotechnology, Donghua University, Shanghai, People’s Republic of China; 2 Department of Biochemistry and Molecular Biology, Dalhousie University, Halifax, Nova Scotia, Canada; University of South Florida, United States of America

## Abstract

Inteins catalyze a protein splicing reaction to excise the intein from a precursor protein and join the flanking sequences (exteins) with a peptide bond. In a split intein, the intein fragments (I_N_ and I_C_) can reassemble non-covalently to catalyze a *trans*-splicing reaction that joins the exteins from separate polypeptides. An atypical split intein having a very small I_N_ and a large I_C_ is particularly useful for joining synthetic peptides with recombinant proteins, which can be a generally useful method of introducing site-specific chemical labeling or modifications into proteins. However, a large I_C_ derived from an *Ssp* DnaX intein was found recently to undergo spontaneous C-cleavage, which raised questions regarding its structure-function and ability to *trans*-splice. Here, we show that this I_C_ could undergo *trans*-splicing in the presence of I_N_, and the *trans*-splicing activity completely suppressed the C-cleavage activity. We also found that this I_C_ could *trans*-splice with small I_N_ sequences derived from two other inteins, showing a cross-reactivity of this atypical split intein. Furthermore, we found that this I_C_ could *trans*-splice even when the I_N_ sequence was embedded in a nearly complete intein sequence, suggesting that the small I_N_ could project out of the central pocket of the intein to become accessible to the I_C_. Overall, these findings uncovered a new atypical split intein that can be valuable for peptide-protein *trans*-splicing, and they also revealed an interesting structural flexibility and cross-reactivity at the active site of this intein.

## Introduction

Inteins are internal protein sequences that can catalyze a protein splicing reaction to excise themselves from a precursor protein and at the same time join the flanking sequences (N- and C-exteins) with a peptide bond [1], [2]. The catalytic mechanism of protein splicing typically consists of four steps [2], [3]: 1) an N-S or N-O acyl rearrangement at the upstream splicing junction replaces the peptide bond with an ester bond; 2) a transesterification reaction transfers the N-extein from the upstream splice junction to the downstream splice junction; 3) an asparagine cyclization at the C-terminus of the intein breaks the peptide bond, which separates the intein from the exteins; and 4) a S-N or O-N acyl rearrangement forms a peptide bond joining the N- and C-exteins. Inteins can sometimes catalyze protein cleavage at their N- or C-termini when the splicing mechanism is disrupted [3]. For example, when step 1 of the splicing mechanism is blocked or missing, step 3 may still occur and break the peptide bond at the C-terminus of the intein, in what is termed C-cleavage. Whereas different inteins show low levels of similarity at the amino acid sequence level, the crystal structures of different inteins (the protein splicing domain) appear very similar [4], [5], [6], [7]. The splicing domains of inteins are typically shaped like a flattened disk consisting of ∼12 β-strands, with the N- and C-terminal parts of the intein folded into a centrally located catalytic pocket.

In a split intein, the intein sequence is broken into two separate fragments, and these intein fragments (I_N_ and I_C_) can reassemble non-covalently to catalyze a protein *trans-*splicing reaction [8], [9]. This reaction takes place between two separate polypeptides, with one polypeptide consisting of an N-extein fused to the I_N_ and the other polypeptide consisting of the I_C_ fused to a C-extein. During *trans*-splicing, the I_N_ and I_C_ are excised, while the N- and C-exteins are concomitantly joined with a peptide bond. Protein-protein *trans*-splicing using split inteins has proven to be a useful technology with a wide range of applications. Examples include production of cytotoxic proteins that cannot be expressed in a single piece [10], [11], segmental isotope labeling of proteins for NMR studies [12], and a gene therapy procedure using split genes [13]. For peptide-protein *trans*-splicing, a non-canonical split intein (*Ssp* DnaB S1) consisting of a small I_N_ and a large I_C_ has been engineered [14]. This atypical split intein is particularly suitable for splicing synthetic peptides onto the N-terminus of recombinant proteins, because the extremely small I_N_ (11 aa long) can be more readily produced together with a small N-extein through chemical synthesis. Because the chemically synthesized N-extein can potentially contain any desired chemical group, this atypical split intein has been used successfully in adding fluorescent labels to the N-terminus of recombinant proteins *in vitro* [15] and on the surface of live mammalian cells [16].

Further development of the intein-based method of protein site-specific modification or labeling is of great interest for protein research and engineering. For example, fluorescent or isotope labels can be useful for studying cellular location and trafficking of proteins, and chemical modifications (e.g. unnatural amino acids) can aid studies of protein's structure-function relationship. Standard chemical methods often produce mixed populations of the modified protein, because such methods usually target certain amino acid side chains (thiols, carboxyls, amines) that may exist at multiple locations in the protein [17]. Other methods have been developed for site-specific protein modifications with limited success. Certain polypeptide tags have been used in recombinant proteins to attach a chemical group through an enzymatic reaction (e.g. [18]), but the tag remains incorporated in the modified protein and may interfere with protein function. Specially engineered tRNA charging systems have been used to add unnatural amino acids to proteins during translation [19], but it is difficult or impossible to engineer special tRNA charging systems for every desired unnatural amino acid or chemical modification. Intein-based protein-peptide *trans*-splicing is a newer and potentially more useful method for site-specific protein modifications [20], [21], [22], because it does not leave a large tag in the modified protein and may be used generally with any chemical moieties on the chemically synthesized extein peptide. To further develop this intein-based method, it is important to find new and atypical split inteins for the peptide-protein *trans*-splicing, because different inteins may exhibit different splicing efficiencies with different exteins [23], [24], [25], [26]. In a recent such study, however, a large C-intein (I_C_) derived from the *Ssp* DnaX intein (a natural intein in DnaX protein of *Synechocystis* sp. PCC6803) was found to undergo spontaneous C-cleavage [27], which is unlike the similarly constructed I_C_ from the *Ssp* DnaB intein (a natural intein in DnaB protein of *Synechocystis* sp. PCC6803). This C-cleavage activity was unexpected, because the large I_C_ without I_N_ was thought to have a structural hole in the catalytic pocket of the intein, based on the predicted structure of the intein. This surprise finding raised interesting questions regarding the structure and function of this intein, namely whether it can still catalyze protein *trans*-splicing in the presence of the I_N_ and, if so, whether its catalytic pocket has unusual structural flexibilities that are not apparent from intein crystal structures.

In this study, we initially found that the unusual I_C_ of the *Ssp* DnaX intein could undergo protein *trans*-splicing with the small I_N_ on another protein, and this *trans*-splicing activity completely predominated over the C-cleavage activity. The I_C_ could also *trans*-splice with small I_N_ sequences derived from other different inteins, which revealed for the first time a cross-reactivity of atypical split inteins. We also found that the small I_N_ could be replaced functionally by a nearly complete intein containing the I_N_. These findings not only generated a new and second atypical split intein suitable for *trans*-splicing peptides onto the N-terminus of proteins, they also have interesting implications for the structure-function of this atypical intein and perhaps also other inteins. We suggest that the centrally located catalytic pocket of the intein might undergo reversible transitions between an open state for the *trans*-splicing function and a closed state for the C-cleavage function, and this structural flexibility might permit the I_N_ part of the intein to swing out of the central pocket of the intein.

## Results

**Figure 1:**
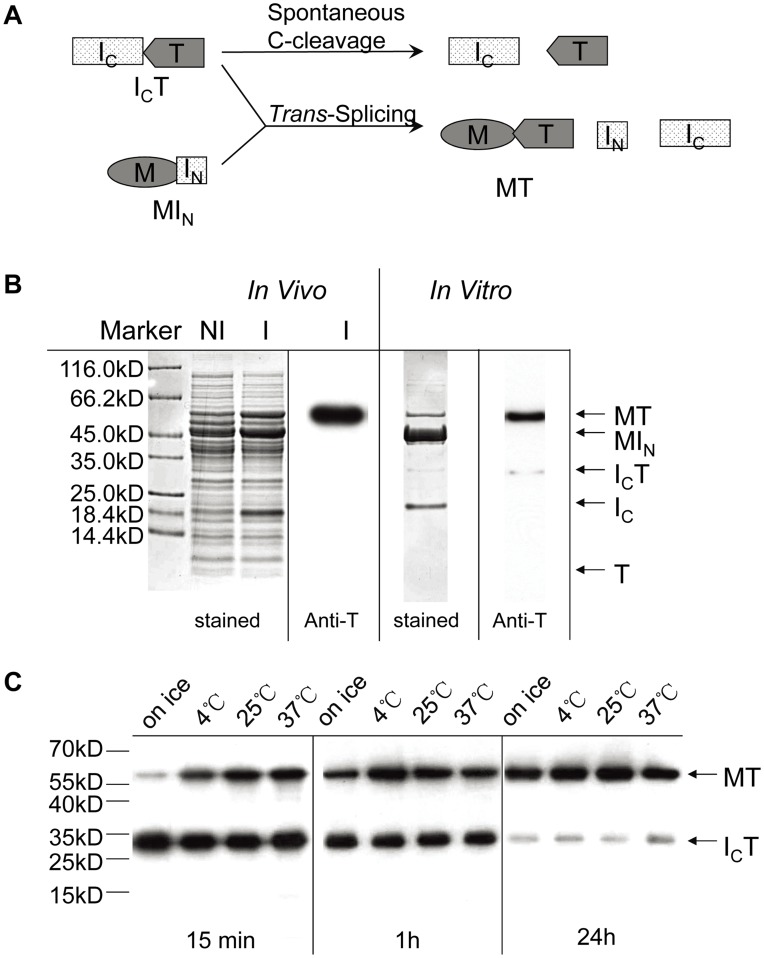
Detection of *trans*-splicing and C-cleavage activities. *A*. Schematic illustration of the C-cleavage and *trans*-splicing reactions. Precursor protein I_C_T is a fusion protein consisting of the 139-aa C-intein (I_C_) of the *Ssp* DnaX split intein fused to a thioredoxin protein (T). Precursor protein MI_N_ is a fusion protein consisting of a maltose binding protein (M) and the 11-aa N-intein (I_N_) of the *Ssp* DnaX split intein. *B*. Experimental analysis of the reactions. For analysis *in vivo*, MI_N_ and I_C_T proteins were co-expressed in *E. coli* cells. Total cellular proteins before (NI) and after (I) the IPTG-induced expression were resolved by SDS-PAGE, and protein bands were visualized either by staining (Coomassie stained) or by Western blotting using an anti-thioredoxin (Anti-T) antibody. For analysis *in vitro*, the MI_N_ and I_C_T proteins were separately produced and purified. These two proteins were then mixed and incubated at room temperature for 20 hours. Reaction products were analyzed and visualized by staining or Western blotting as above. Positions are indicated for the precursor proteins (MI_N_ and I_C_T), the splicing product (MT), and the C-cleavage products (I_C_ and T). Size markers (Marker) are shown on the left. *C.* Effects of reaction times and temperatures. The *in vitro* reactions were carried out for the specified length of times and at the specified temperatures. Reaction products were analyzed by Western blotting as above.

First we determined whether the 139-aa C-intein (I_C_) of the *Ssp* DnaX intein could undergo protein *trans*-splicing when the missing 11-aa N-terminal part (N-intein or I_N_) was provided *in trans,* because the I_C_ alone had been found to undergo spontaneous C-cleavage [27]. As illustrated in Figure 1A, a maltose binding protein (M) and a thioredoxin protein (T) were used as the N-extein and C-extein, respectively, so that a *trans*-splicing reaction would join these two exteins to form the splicing product MT. As seen in Figure 1B, the splicing product MT was produced both *in vivo* when the two precursor proteins (MI_N_ and I_C_T) were co-expressed in *E. coli* and *in vitro* when the purified precursor proteins were incubated together in a test tube.

Interestingly, no C-cleavage activity was detected under these conditions, as indicated by an absence of the cleavage product T. The precursor and product proteins were identified by their predicted sizes and specific recognition of an anti-thioredoxin (anti-T) antibody through Western blotting. For the *in vivo* analysis in *E. coli* cells, only the splicing product MT protein was detected using anti-T antibody, indicating that the precursor protein I_C_T had *trans*-spliced completely to form the MT protein. For the *in vitro* analysis, the purified precursor protein MI_N_ was added in excess to the precursor protein I_C_T to drive the *trans*-splicing reaction to greater completion. In producing the I_C_T protein alone in *E. coli*, a significant amount of spontaneous C-cleavage occurred as reported previously [27]. In the purification of I_C_T using an affinity tag (hexahistidine) contained in the I_C_, the cleavage product I_C_ was co-purified with the remaining I_C_T in the purified sample, while the cleavage product T lacked the hexahistidine tag and was absent in the purified sample. The purified I_C_T protein did not show new C-cleavage during subsequent co-incubation with the MI_N_ protein for *trans*-splicing, as indicated by the absence of any new formation of the C-cleavage products I_C_ and T. Under the *in vitro* conditions used, approximately 85% of the I_C_T protein was *trans*-spliced to form the MT protein after 20 hours of incubation at room temperature (Figure 1B). We also tested shorter reaction times and different temperatures (Figure 1C). The efficiency of *trans*-splicing was nearly identical at four tested temperatures (4, 25, and 37°C, and on ice) after 24 hours of reaction. With a shorter reaction time of 15 minutes, the efficiency of *trans*-splicing was a little lower at 4°C and significantly lower on ice.

**Figure 2:**
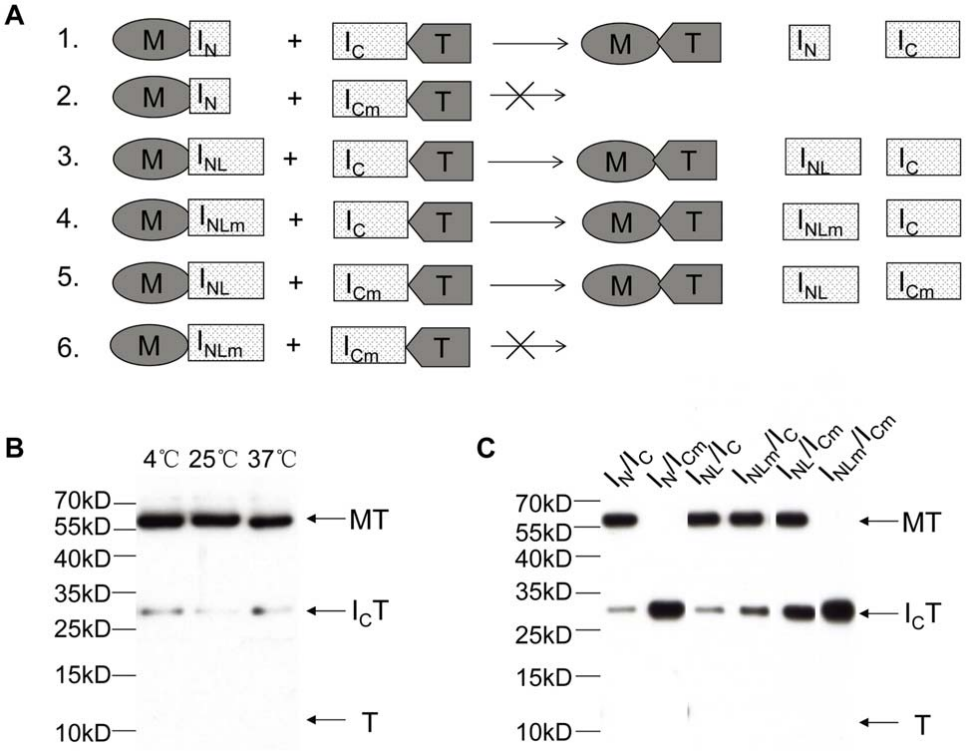
Effects of I_N_ size and Block B mutation on *trans*-splicing. *A*. Schematic illustration of experimental designs. I_NL_ is the N-terminal 144-aa sequence of the *Ssp* DnaX intein. I_NLm_ and I_Cm_ are the same as I_NL_ and I_C_, respectively, except that the conserved Block B sequence of the intein was mutated from TXXH to AXXA. Others are the same as in Figure 1A. *B*. *Trans*-splicing of I_C_T with MI_NL_. A mixture of the two precursor proteins was incubated at the specified temperatures for 20 hours to allow reaction, with the protein bands visualized by Western blotting using anti-T antibody. *C*. *Trans*-splicing of different precursor protein pairs listed in panel A. Each pair of precursor proteins was incubated together at room temperature for 20 hours, with the protein bands visualized by Western blotting using anti-T antibody. Names of only intein parts of the precursor proteins are specified on the top of the panel.

We then investigated whether the *trans*-splicing reaction could still occur when the small I_N_ is embedded in a near complete intein. As illustrated in Figure 2A, the intein fragment I_NL_ was designed to contain the N-terminal 144-aa sequence of the 150-aa *Ssp* DnaX intein that lacked the C-terminal 6 aa of the intein, to prevent possible self-cleavage or *cis*-splicing. This I_NL_ was found to *trans*-splice efficiently with I_C_ *in vitro* at three different temperatures (4, 25, and 37°C), where M and T were the exteins (Figure 2B). Because the 144-aa I_NL_ consists of the small (11-aa) I_N_ plus other parts of the intein, we asked whether the other parts of the intein also participated in the *trans*-splicing reaction. To answer this question, a double mutation (TXXH to AXXA) was introduced in the Block B motif of the intein, because this conserved intein sequence motif is outside the 11-aa I_N_ and known to be functionally important in inteins [25]. As seen in Figure 2C, mutating the Block B motif of I_C_ (resulting in I_Cm_) destroyed its ability to *trans*-splice with I_N_, as expected. In contrast, mutating the Block B motif of I_NL_ (resulting in I_NLm_) did not affect its ability to *trans*-splice with I_C_, indicating that the Block B motif in I_NL_ did not participate in the reaction. We also tested mutated C-intein (I_Cm_) in a combination with the non-mutated version of I_NL_, and found that latter could compensate for the mutated Block B motif in the former for the *trans*-splicing reaction. When the Block B motif was mutated in both I_NL_ (resulting in I_NLm_) and I_C_ (resulting in I_Cm_), the *trans*-splicing reaction was abolished, as expected.

**Figure 3:**
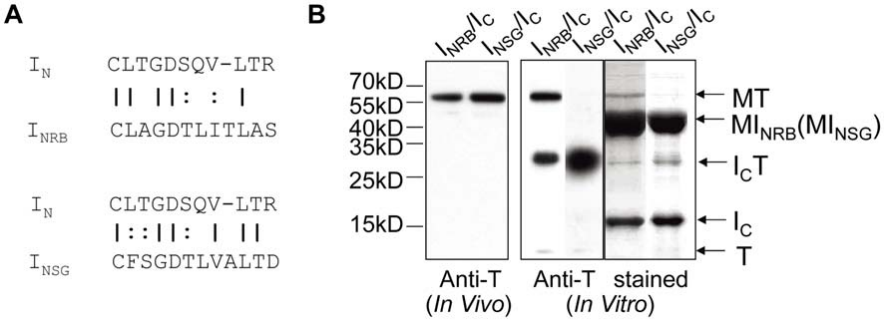
Cross-reactivity of I_C_ with I_N_ from different inteins. *A.* Comparison of the amino acid sequences of I_N_ from *Ssp* DnaX intein (I_N_), *Rma* DnaB intein (I_NRB_), and *Ssp* GyrB intein (I_NSG_). Identical and similar residues are marked with a | and a :, respectively. A gap (represented with a -) is introduced in the I_N_ sequence to maximize the sequence alignment. *B*. Analysis of *trans*-splicing reactions between I_C_ and I_NRB_ (or I_NSG_) as specified on top. For *in vivo* analysis, MI_NRB_ (or MI_NSG_) and I_C_T proteins were co-expressed in *E. coli* cells, and total cellular proteins were analyzed by Western blotting using an anti-thioredoxin (Anti-T) antibody. For *in vitro* analysis, purified MI_NRB_ (or MI_NSG_) and I_C_T proteins were co-incubated at room temperature for 20 hours, and the reaction products were analyzed by Western blotting as above. Positions are marked for the precursor I_C_T, splicing product MT, and C-cleavage product T. Size markers are shown on the left.

To further explore the structural flexibility and versatility of this atypical split intein, we asked whether the large I_C_ could *trans*-splice with small I_N_ sequences derived from other inteins. The 12-aa I_NRB_ was derived from the N-terminus of the *Rma* DnaB intein (a natural intein in DnaB protein of *Rhodothermus marinus*) [28] that is highly similar to the *Ssp* DnaB intein from which the first atypical split intein was derived [13]. The 12-aa I_NSG_ was derived from the N-terminus of the *Ssp* GyrB intein (a natural intein in GyrB protein of *Synechocystis* sp. PCC6803) [29]. As shown in Figure 3A, the I_NRB_ sequence is 41% identical (58% similar) to the I_N_ sequence, and the I_NSG_ sequence is 50% identical (75% similar) to the I_N_ sequence. Under *in vivo* conditions in *E. coli*, both I_NRB_ and I_NSG_ *trans*-spliced efficiently with I_C_, as indicated by the accumulation of splicing product MT but not precursor protein I_C_T (Figure 3B). Under *in vitro* conditions using purified precursor proteins, I_NRB_ *trans*-spliced with I_C_, but I_NSG_ did not. For I_NRB,_ the *in vitro trans*-splicing reaction did not go to completion, with ∼60% of the precursor protein I_C_T remaining. This was not due to a lesser amount of I_NRB_, because Coomassie-stained gel pictures showed an excess amount of the precursor protein MI_NRB_ in the reaction (Figure 3B). This may indicate an inefficient use of I_NRB_ under the *in vitro* conditions used, although an efficient use of I_NRB_ was seen in *E. coli* cells. With both I_NRB_ and I_NSG,_ the precursor protein I_C_T underwent a small amount of C-cleavage, as indicated by the accumulation of a small amount of the C-cleavage product T.

## Discussion

**Figure 4:**
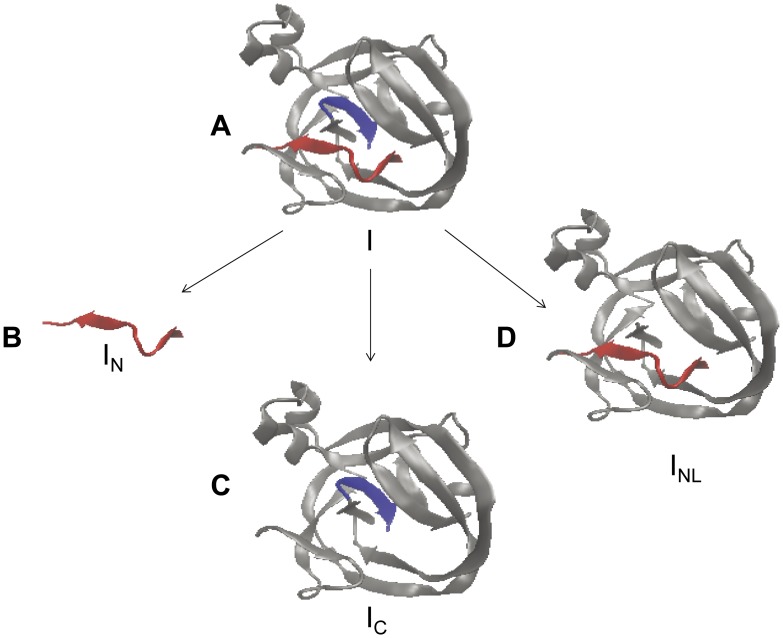
Structural modeling of the intein and its parts. Computer-based modeling of the *Ssp* DnaX intein (A) and its parts (B, C, D) used the VMD program (http://www.ks.uiuc.edu/Research/vmd/) and the crystal structure of the *Ssp* DnaB mini-intein [7]. The 11-aa I_N_ part of the intein is shown in red. The C-terminal 6-aa part of the intein is shown in blue.

The atypical *Ssp* DnaX intein is found, for the first time, to be capable of protein *trans*-splicing, despite the fact that its large C-intein (I_C_) had been known to undergo spontaneous C-cleavage. This finding has interesting implications on the structure-function of inteins’ active site. Previously the I_C_ part of this intein was found to undergo spontaneous C-cleavage in the absence of I_N_ [27], which was quite unexpected and unlike other inteins. The highly conserved crystal structures of inteins predict that the N- and C-terminal parts of an intein are located in a central catalytic pocket [5], [6], [7], as illustrated by a computer modeling of the *Ssp* DnaX intein shown in Figure 4A. The 11-aa I_N_ sequence forms two small β-strands named β1 and β2, with β1 being buried deep inside the intein structure. Without I_N_, the I_C_ structure has been predicted to have a structural void (hole) in its catalytic pocket [27], as illustrated in Figure 4C. A similar prediction has also been made for I_C_ of the *Ssp* DnaB intein [30], where the hole was thought to be a docking place for the I_N_ to trigger a C-cleavage reaction. To explain why the I_C_ of *Ssp* DnaX intein (but not of *Ssp* DnaB intein) could undergo spontaneous C-cleavage in the absence of I_N_, it was suggested that the predicted hole of this I_C_ might be sealed or compensated to allow the spontaneous C-cleavage [27]. Our findings in this study show that this I_C_ can also perform protein *trans*-splicing with I_N_, indicating that the predicted hole of I_C_ is open at least some of the time, in order for the I_N_ to dock for *trans*-splicing. We suggest that the hole of I_C_ may exist in two equilibrium states: an open state (hole is open) allowing the docking of I_N_ for *trans*-splicing, and a closed state (hole is closed) allowing spontaneous C-cleavage without I_N_. Our findings also indicate that the open state predominates, because the *trans*-splicing reaction completely suppressed the C-cleavage reaction when I_N_ was present. Our suggestion is consistent with an earlier study of the *Ssp* DnaB intein, where the horseshoe-like structure of the large I_C_ was suggested to open up and clamp onto the small I_N_ [30].

The above suggestion is further supported by our finding that the *trans*-splicing reaction could occur when the small I_N_ was replaced with I_NL_, where I_NL_ is a near complete intein containing the I_N_. The I_N_ part of I_NL_ must have participated in the *trans*-splicing reaction, whereas the remaining part of I_NL_ (at least the conserved Block B motif) apparently did not participate in the reaction, because a mutation in the Block B motif of I_NL_ did not prevent *trans*-splicing. To participate in the *trans*-splicing reaction, the I_N_ part of I_NL_ needs to move out from its buried position in I_NL_ (Figure 4D), before it can dock into the catalytic pocket (open hole) of I_C_ for *trans*-splicing. This suggests that the I_NL_ structure is also able to open up in order for the I_N_ part to ‘swing out’ to an exposed position. Consistent with this suggestion, a mutated C-intein (I_Cm_) *trans*-spliced with I_NL_, suggesting that the I_Cm_ structure could open up to allow its C-terminal part to “swing out” to an exposed position for participation in the *trans*-splicing reaction. Furthermore, we discovered cross-reactivity between I_C_ of the atypical *Ssp* DnaX split intein and the small I_N_ from two other inteins, which is the first finding of cross-reactivity for such atypical split inteins. Considering that the I_N_ of the other inteins is only 40–50% identical to the native I_N_ in amino acid sequence, it is interesting that the non-native I_N_ can correctly dock into the structural hole of the I_C_ and catalyze the *trans*-splicing reaction. Overall, these findings revealed an interesting structural flexibility at or near the catalytic pocket of inteins, which can have significant implications on future engineering of split inteins for peptide-protein or protein-protein *trans*-splicing for various applications.

The *trans*-splicing function of this atypical *Ssp* DnaX split intein also makes a significant addition to the intein-based toolbox for general uses. Previously only the *Ssp* DnaB intein has been engineered into such an atypical split intein, and was named the S1 split intein [14]. Unlike other forms of split inteins, the S1 split intein has an extremely small I_N_ and is therefore particularly useful for splicing synthetic peptides onto the N-terminus of target proteins. The synthetic peptide can easily accommodate the 11-aa I_N_ plus a small N-extein to be spliced onto the N-terminus of a target protein, with the target protein being a fusion protein containing the I_C_. This peptide-protein *trans*-splicing is useful for site-specific labeling or modifications of proteins, because the synthetic N-extein may be engineered to carry a variety of chemical moieties, including fluorescent groups, modified or unnatural amino acids, and drug molecules, as long as the chemical moiety does not block *trans*-splicing. Finding and understanding new S1 split inteins, as we have done in this study, is important for wide uses of this peptide-protein *trans*-splicing method, because different inteins have been known to splice differently when used on different target proteins [25], [31]. It is impressive that this new S1 split intein could perform the *trans*-splicing reaction at temperatures ranging from ∼1°C (on ice) to 37°C, although the reaction speed was somewhat lower at 1–4°C temperatures. This temperature tolerance may be due to the fact that this intein was derived from a natural intein found in a cyanobacterium (*Syenochocystis* sp. PCC6803) that lives under a wide range of environmental temperatures. This robust nature of the S1 split intein can be an advantage in practical applications where one may need to achieve *trans*-splicing under low temperatures. Our finding of cross-reactivity between the I_C_ of the atypical *Ssp* DnaX split intein and the small I_N_ from two other inteins also has interesting implications. On the one hand, it permits different choices for the I_N_ for doing peptide-protein *trans*-splicing and suggests that the I_N_ sequence may tolerate many sequence changes, which can be useful information for designing and producing synthetic peptides containing I_N_. On the other hand, two atypical split inteins may not be used together in a mixed system to achieve labeling or modification of two different target proteins in a protein-specific manner.

## Materials and Methods

### Plasmid Construction

Plasmid pMSX-S1 for *in vivo* experiments was constructed as described previously [14], in which the two open reading frames expressing the MI_N_ and I_C_T proteins were separated by a spacer sequence. A restriction enzyme cutting site Afl II was introduced at the split site. To construct plasmid pMSX-S1N expressing the MI_N_ protein alone, a DNA fragment between Afl II and Hind III sites was deleted from plasmid pMSX-S1. Plasmid pMSX-S1C expressing the I_C_T protein alone was from [27]. Plasmid pMSX-S1NL expressing the MI_NL_ protein was constructed by replacing the I_N_ coding sequence in pMSX-S1N with the first 141 codons of the *Ssp* DnaX intein [32], which used standard recombinant DNA methods including PCR, DNA cutting(XhoI- Afl II), and ligation. Plasmid pMRB-S1N expressing the MI_NRB_ protein and plasmid pMSG-S1N expressing the MI_NSG_ protein were constructed by replacing the I_N_ coding sequence in pMSX-S1N with the first 12 codons of the *Rma* DnaB intein [28] and *Ssp* GyrB intein [29], respectively. To construct pMRBSX-S1, pMSGSX-S1, the I_N_ coding sequence between XhoI and Afl II in pMSX-S1was replaced with I_NRB_ or I_NSG_ sequence respectively. To construct plasmid pmMSX-S1C (or pmMSX-S1NL) expressing the I_Cm_T (or MI_NLm_) protein, site-directed mutations were introduced into plasmid pMSX-S1C (or pMSX-S1NL), using a standard method of inverse PCR.

### Protein Expression, Purification and in vitro Reactions

Plasmids pMSX-S1, pMSX-S1N, pMSX-S1NL, pMRB-S1N, pMSG-S1N and pmMSX-S1NL were each transformed into *Escherichia coli* DH5α cells, while plasmids pMSX-S1C and pmMSX-S1C were each transformed into *Escherichia coli* BL21(DE3) cells, all using a standard *E. coli* transformation protocol. The transformed *E. coli* cells were grown in 50 mL of Luria Broth (LB) medium at 37°C to mid-logarithmic phase (OD_600_ of ∼0.6) and induced by 0.8 mM IPTG to express, at room temperature overnight, the plasmid-encoded protein(s) of interest. To analyze total cellular proteins, cells were harvested by centrifugation and solubilized directly in SDS loading buffer. For protein purification, harvested cells were lysed using a French Press (14,000 PSI), and the cell lysate was centrifuged to remove any insoluble materials. To purify the MI_N_, MI_NL_, and MI_NLm_ proteins containing the maltose binding protein, amylose resin was used according to the manufacturer’s instructions (New England Biolabs). To purify the I_C_T and I_Cm_T proteins containing a hexahistidine tag, Ni-NTA resin (QIAGEN) was used according to the manufacturer’s instructions. For *in vitro trans*-splicing or cleavage reactions, the specified precursor proteins were mixed and incubated under specified conditions, with 1 mM DTT added to all *in vitro* reactions. Western blotting used an anti-thioredoxin (anti-T) antibody (Invitrogen) and the Enhanced Chem-luminescence detection kit (GE Healthcare), all according to the manufacturer’s instructions.

### Computational Simulations of Intein Structure

A simulated three-dimensional structure of *Ssp* DanX mini-intein was obtained by using the fully automated homology-modeling pipeline SWISS-MODEL [33], [34]. The intein amino acid sequence [27] was uploaded to the Automatic Modeling Workspace (http://swissmodel.expasy.org/workspace), the crystal structure of *Ssp* DnaB mini-intein was selected automatically as a closest template by homology, and structural models of *Ssp* DanX mini-intein were generated and presented in NewCartoon style.
